# Brain structural connectome in relation to PRRT2 mutations in paroxysmal kinesigenic dyskinesia

**DOI:** 10.1002/hbm.25091

**Published:** 2020-06-27

**Authors:** Lei Li, Du Lei, Xueling Suo, Xiuli Li, Chen Yang, Tianhua Yang, Jiechuan Ren, Guangxiang Chen, Dong Zhou, Graham J. Kemp, Qiyong Gong

**Affiliations:** ^1^ Huaxi MR Research Center (HMRRC), Department of Radiology West China Hospital of Sichuan University Chengdu Sichuan Province China; ^2^ Department of Psychiatry and Behavioral Neuroscience University of Cincinnati College of Medicine Cincinnati Ohio USA; ^3^ Department of Neurology West China Hospital of Sichuan University Chengdu Sichuan Province China; ^4^ Department of Neurology Beijing Tiantan Hospital, Capital Medical University Beijing China; ^5^ Department of Radiology The Affiliated Hospital of southwest Medical University Luzhou China; ^6^ Liverpool Magnetic Resonance Imaging Center (LiMRIC) and Institute of Life course and Medical Sciences University of Liverpool Liverpool UK; ^7^ Psychoradiology Research Unit of Chinese Academy of Medical Sciences, Functional and Molecular Imaging Key Laboratory of Sichuan Province West China Hospital of Sichuan University Chengdu Sichuan China

**Keywords:** connectome, diffusion tensor imaging, mutation, paroxysmal kinesigenic dyskinesia, psychoradiology

## Abstract

This study explored the topological characteristics of brain white matter structural networks in patients with Paroxysmal Kinesigenic Dyskinesia (PKD), and the potential influence of the brain network stability gene *PRRT2* on the structural connectome in PKD. Thirty‐five PKD patients with *PRRT2* mutations (PKD‐M), 43 PKD patients without *PRRT2* mutations (PKD‐N), and 40 demographically‐matched healthy control (HC) subjects underwent diffusion tensor imaging. Graph theory and network‐based statistic (NBS) approaches were performed; the topological properties of the white matter structural connectome were compared across the groups, and their relationships with the clinical variables were assessed. Both disease groups PKD‐M and PKD‐N showed lower local efficiency (implying decreased segregation ability) compared to the HC group; PKD‐M had longer characteristic path length and lower global efficiency (implying decreased integration ability) compared to PKD‐N and HC, independently of the potential effects of medication. Both PKD‐M and PKD‐N had decreased nodal characteristics in the left thalamus and left inferior frontal gyrus, the alterations being more pronounced in PKD‐M patients, who also showed abnormalities in the left fusiform and bilateral middle temporal gyrus. In the connectivity characteristics assessed by NBS, the alterations were more pronounced in the PKD‐M group versus HC than in PKD‐N versus HC. As well as the white matter alterations in the basal ganglia‐thalamo‐cortical circuit related to PKD with or without *PRRT2* mutations, findings in the PKD‐M group of weaker small‐worldness and more pronounced regional disturbance show the adverse effects of *PRRT2* gene mutations on brain structural connectome.

## Graphical abstract

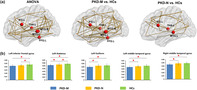

## 1 INTRODUCTION

Paroxysmal kinesigenic dyskinesia (PKD) is a rare movement disorder most commonly inherited in autosomal dominant mode (Bruno et al., 2004), with a reported prevalence of 1:150,000 (W. J. Chen et al., 2011; Spacey & Adams, 1993). It is characterized by recurrent, transient, unilateral or bilateral, dystonic or choreoathetoid attacks triggered by sudden voluntary movements (e.g., change of position or velocity, or shock), with preserved consciousness (Bhatia, 1999; Bruno et al., 2004; Demirkiran & Jankovic, 1995), which last from a few seconds to a few minutes, and can be rare (once a month) or frequent (up to 100 times per day). The age of onset ranges from infancy to late middle age, most commonly in childhood and adolescence (Spacey & Adams, 1993). The disease typically responds well to antiepileptic drugs (Bhatia, 1999; Demirkiran & Jankovic, 1995), but it carries a high psychological burden, and impairs quality of life (Tian et al., 2017); it is often clinically misdiagnosed as epilepsy (Ebrahimi‐Fakhari et al., 2014). The underlying pathophysiological mechanisms in PKD are not fully understood despite some recent successes using neuroimaging to define structural and functional brain abnormalities mainly in the basal ganglia‐thalamo‐cortical circuits (Joo et al., 2005; J. H. Kim, Kim, Kim, Suh, & Koh, 2015; Ko, Kong, Ngai, & Ma, 2001; Long et al., 2017; Monge‐Argiles, Bautista‐Prados, Perez‐Vicente, & Morales‐Ortiz, 2001; Shirane, Sasaki, Kogure, Matsuda, & Hashimoto, 2001; Zhou, Chen, Gong, Tang, & Zhou, 2010; Zhou, Chen, Zhang, et al., 2010), as well as other cerebral cortical areas (Luo et al., 2013; Ren et al., 2015).

Recently the *PRRT2* gene, encoding the proline‐rich transmembrane protein 2, has been identified as the causative gene for PKD (W. J. Chen et al., 2011; J. L. Wang et al., 2011), and is useful for diagnosis and as a clue to the pathogenesis. PKD patients with *PRRT2* mutations (PKD‐M) show distinct clinical manifestations including earlier age of onset and longer duration of attacks, complex phenotypes of dystonia and chorea, and more tendency for a family history compared to PKD patients without *PRRT2* mutations (PKD‐N) (Huang et al., 2015). Neuroimaging studies have provided evidence of various brain effects of *PRRT2*: for example, thalamo‐prefrontal hypoconnectivity (Long et al., 2017) and increased functional activity in the right postcentral gyrus (Luo et al., 2013) in PKD‐M compared to PKD‐N patients. *PRRT2* is highly expressed in the developing nervous system (W. J. Chen et al., 2011). It consists of four exons encoding the PRRT2 protein with two transmembrane domains; the truncating mutations alter the subcellular localization of its protein, resulting in a complete loss of its transmembrane ion channel function that leads to clinical manifestations of PKD such as dystonic or choreoathetoid attacks (W. J. Chen et al., 2011).

*PRRT2* is considered a brain network stability gene, loss of whose function leads to changes in the short‐term plasticity dynamics of excitatory synapses; this impairs the functional stability of neuronal networks, rendering them more susceptible to externally‐triggered paroxysmal events (Michetti, Corradi, & Benfenati, 2017). A whole‐brain structural network methodology is therefore a promising new perspective from which to study the role of *PRRT2* in PKD. Going beyond the study of individual brain areas or seed‐based connectivity, a connectome‐based approach considers the whole brain as a set of interconnected networks (Sporns, Tononi, & Kotter, 2005). Graph theoretical analysis (Fornito, Zalesky, & Breakspear, 2013) based on diffusion tensor imaging (DTI) data can be used to explore white matter structural network topology (Bullmore & Sporns, 2009; Gong, He, et al., 2009; Suo, Lei, Chen, et al., 2017; Suo et al., 2019; Zhao et al., 2015). The normal human connectome functions as a “small‐world” network pattern of high efficiency, characterized by high global integration (e.g., low characteristic path length) and high local specialization (e.g., high clustering coefficient; Watts & Strogatz, 1998). DTI‐based connectome methods have identified white matter structural network topological abnormalities in many psychiatric and neurological disorders, and have proved an effective way to explore pathophysiological mechanisms and investigate potential disease biomarkers (Li et al., 2017; Suo, Lei, Chen, et al., 2017; Suo et al., 2018; Suo et al., 2019; Q. Wang et al., 2012). Using DTI‐based structural connectome methods to study whole‐brain white matter structural networks in PKD patients with and without *PRRT2* gene mutation may give insights into the anatomical basis of the effects of *PRRT2* modulation at the network level.

To this end, we set out to evaluate the effect of *PRRT2* on brain structural connectome in PKD by performing DTI studies on 35 PKD‐M patients, 43 PKD‐N patients, and 40 demographically‐matched healthy controls (HCs). We used a graph theoretical analysis to identify alterations in the topological properties of brain white matter structural networks in PKD‐M patients versus PKD‐N patients and HCs. We also conducted a subgroup analysis on drug‐naive PKD‐M and PKD‐N patients to eliminate the potential confounding effects of medication on structural connectome alterations. We tested two hypotheses. First, given the important role of *PRRT2* in brain network stability, we hypothesized that *PRRT2* mutations would adversely affect brain white matter structural networks in PKD, independently of the potential effects of medication. Second, considering reports of decreased white matter integrity in the thalamic radiation and volume changes in the thalamus in PKD, we predicted corresponding nodal abnormalities in the present study.

## 2 MATERIALS AND METHODS

### 2.1 Participants

Seventy‐eight patients with PKD were recruited via the neurology department of West China Hospital of Sichuan University, Chengdu. The diagnosis of PKD was made using the criteria proposed by (Bruno et al., 2004). The inclusion criteria for the patients were: (a) identified kinesigenic triggers for attacks; (b) attacks of short duration (<1 min); and (c) no loss of consciousness or pain sensation during attacks. The exclusion criteria for all participants were: (a) presence of focal brain lesions on routine magnetic resonance imaging (MRI); (b) any standard MRI incompatibility; (c) history of alcohol/substance abuse; and (d) comorbidity with neurological or psychiatric disorders or other serious physical diseases. This study was approved by West China Hospital of Sichuan University research ethics committee, and written informed consent was obtained from all participants or their legal guardians prior to study participation.

Forty age‐ and sex‐ matched HC participants were recruited from the local area by poster advertisements and examined by experienced neurologists; participants were excluded from the study if they had any neurological illness, as assessed by clinical evaluation and medical records.

### 2.2 Genetic analysis

In patients with PKD, genomic DNA was isolated from the peripheral blood using a standard phenol/chloroform extraction procedure. *PRRT2* (NM_145239) mutations were identified by Sanger sequencing procedure using an ABI 3730 automated DNA sequencing system (Invitrogen, Shanghai, China) under standard conditions. Briefly, the polymerase chain reaction (PCR) products were first amplified using previously published PCR primers specifically designed to amplify the entire exons and the intron‐exon boundaries of *PRRT2* gene (W. J. Chen et al., 2011). Second, the PCR products were purified in 5 μl total volume for one cycle of 60 min at 37°C and 15 min at 80°C. The purified PCR products were treated with the ABI PRISM Big Dye Terminator Cycle Sequencing Ready Reaction sequencing kit. Next, the sequencing products were purified again by ethanol/EDTA/sodium acetate precipitation. Finally, the products were analyzed on the ABI 3730 automated DNA sequencer. Comparing the products' DNA sequence with the genomic DNA sequence of *PRRT2*, *PRRT2* (NM_145239) mutations were identified by numbering the nucleotide change positions that correspond to their positions in *PRRT2* mRNA. Of the 78 patients with PKD, 35 were PKD‐M and 43 PKD‐N.

### 2.3 MRI data acquisition

All scans were performed on 1 Siemens Trio Tim 3T MRI (Siemens, Erlangen) using an 8‐channel phased‐array head coil. Subjects were instructed to relax, keep eyes closed but without falling asleep, and hold still to minimize the head motion. The head was stabilized with cushions, and ear plugs were used. DTI images were acquired using a spin‐echo echo‐planar image based sequence with the following scan parameters: 64 diffusion directions with *b* = 1,000 s/mm^2^ and a reference image without diffusion weighting (*b* = 0), 3 mm slice thickness with no interslice gap, repetition time /echo time (TR/TE) = 6800/91 ms, field of view (FOV) = 19200 mm × 1920 mm, flip angle = 90°, voxel = 0.94 × 0.94 × 3.0 mm^3^ and number of excitations = 2. High resolution T1‐weighted images were obtained using a three‐dimensional (3‐D) spoiled gradient‐recalled sequence with the following scan parameters: 176 axial slices, TR/TE = 1900/2.26 ms, voxel size = 1 × 1 × 1 mm^3^, FOV = 256 × 256 mm^2^, slice thickness = 1 mm, without interslice gap and flip angle = 9°. The absence of gross brain abnormalities was confirmed in all subjects by experienced neuroradiologists using conventional MRI protocols of axial T1‐weighted, T2‐weighted, and fluid‐attenuated inversion recovery images.

All the raw DTI and 3‐D T1‐weighted data were carefully inspected to ensure that no subject with conspicuous head motion or signal dropout was included. We calculated the head motion from the DTI data to exclude subjects with head movements of displacement >2 mm, or translation in the *x*, *y*, or *z* directions, or rotation >2° around the *x*, *y*, or *z* axes. The quantitative values for head motion did not differ between patients and controls (details in Table S1). Dystonic or choreoathetoid attacks in PKD are usually triggered by sudden voluntary movements such as standing up or changing velocity, so do not usually occur during MRI scans. Patients preserve consciousness during attacks, so we explicitly checked for dystonic or choreoathetoid attack by questioning the patients after completing the scan. If an attack had been reported the patient would have been rescanned later; however, none were reported.

### 2.4 Data preprocessing

Structural images were preprocessed using PANDA (http://www.nitrc.org/projects/panda/), a pipeline toolbox for analyzing brain diffusion images using a number of processing functions from FMRIB Software Library (FSL; Cui, Zhong, Xu, He, & Gong, 2013; Smith et al., 2004) implemented in Matlab (Mathworks, Natick, MA; www.mathworks.com). The steps are briefly introduced here. First, all the DICOM files were converted to NIFTI images. The brain extraction tool (BET) was used for skull stripping. Eddy‐current correction was performed for any distortions of diffusion‐weighted images induced by head movements. The DTIFIT command of FSL was used to build the voxel‐wise DTI models to create the fractional anisotropy (FA) maps. These were registered to the Oxford Center of Brain FA template in Montreal Neurological Institute (MNI) space using nonlinear transformation with a voxel size of 2 × 2 × 2 mm^3^.

### 2.5 DTI‐based structural network construction

#### 2.5.1 Node definition

The key to constructing the structural connectome is to define the nodes and edges, the basic elements of a network (Sporns et al., 2005). We used the deterministic tractography in PANDA software (Cui et al., 2013). For each subject, the automated anatomic labeling (AAL) atlas (Tzourio‐Mazoyer et al., 2002) was used for both cortical and subcortical parcellations, resulting in 90 cortical and subcortical regions of interest (45 for each hemisphere); each region representing a network node. This parcellation procedure was chosen, as previously (Gong, He, et al., 2009; Gong, Rosa‐Neto, et al., 2009), because it is the widely used atlas in the graph theory literature. Each individual T1‐weighted image was co‐registered to the b0 image in the DTI native space using a linear transformation, and then mapped to the T1 template in the MNI space using a nonlinear transformation. The derived transformation parameters were inverted and used to warp the AAL mask from the MNI space to the DTI native space, using the nearest neighbor interpolation method to preserve the discrete labeling values in the DTI native space. For quality control, we performed skull‐removal manually for each individual T1‐weighted image, using BET in the FSL.

#### 2.5.2 Edge definition

To define the network edges, the DTI deterministic tractography was performed to reconstruct the whole‐brain white matter tracts by using the Fiber Assignment Continuous Tracking (FACT) algorithm (Mori, Crain, Chacko, & van Zijl, 1999; Mori & van Zijl, 2002). Briefly, the structural T1‐weighted image was first parcellated into the gray matter, white matter, and cerebrospinal fluid, and all the white matter voxels were selected as seed voxels for fiber tracking. The assumption is that the orientation of the largest component of the diffusion tensor represents the orientation of the dominant fiber tract in each voxel. The deterministic fiber tracking procedure advanced along its fiber tract orientation, and was terminated when either FA was <0.2 or turn angle >45° (Mori & van Zijl, 2002). This default FA threshold (angle threshold 45°, FA threshold range 0.2–1) was used to exclude voxels in the gray matter and CSF during the fiber tracking procedure. Fiber tracts were determined by their capacity to link every pair of cortical regions using the refined cortical ALL masks; the pairs of cortical regions were considered anatomically connected. The connection represented an edge in the structural brain network. Thus, an average FA‐weighted symmetrical anatomical 90 × 90 network matrix was obtained for each subject. A white matter network matrix weighted by fiber number (FN) was also created for each subject. The total number of connected fibers between the two regions was defined as the weights of the network edges by computing the sum of all the existing streamline connections that could provide information on the quantity of white matter connectivity between these two regions. The average FA‐weighted matrices were used for the network metric analysis described below. The results of network analysis using the FN‐weighted and FN*FA‐weighted matrices and their binarization (reflecting the existence or absence of connection without accounting for connectivity strength) are given in Supporting Information (Table S2, with an example of the FA, FN, and FA*FN matrices in one subject in Figure S1).

### 2.6 Network analysis

#### 2.6.1 Small‐world properties and network efficiency

Network metric calculations based on graph theoretical analyses were carried out using the GRETNA toolbox (J. Wang et al., 2015) (http://www.nitrc.org/projects/gretna/), which investigated the topological properties of the white matter structural networks at both global and nodal levels. The global‐level graph metrics (Watts & Strogatz, 1998) included the clustering coefficient (*C*_p_), characteristic path length (*L*_p_; Newman, 2003), normalized clustering coefficient (*γ*), normalized characteristic path length (*λ*), small‐worldness scalar (*σ*), local efficiency (*E*_loc_), and global efficiency (*E*_glob_; Latora & Marchiori, 2001). The nodal‐level properties included nodal betweenness, nodal efficiency, and nodal degree (Rubinov & Sporns, 2010).

#### 2.6.2 Threshold selection

We applied a range of network sparsity thresholds (S) to the correlation matrices. Consistent with previous studies (Y. Chen et al., 2019; Lei et al., 2015; Suo et al., 2019; Zhang et al., 2011), the area under the curve (AUC) was calculated over the sparsity range of 0.10 < S < 0.34 with an interval of 0.01. These minimum and maximum values of S ensure that the thresholded networks were estimable for small‐worldness with sparse properties, and had the minimum number of spurious edges (Watts & Strogatz, 1998). The AUC provides a summarized scalar metric for the topological characterization of the brain networks, independent of any specific cost threshold selection and therefore free of the potential bias of any single threshold (Suo, Lei, Li et al., 2017; Zhang et al., 2011). Besides AUC, the graph‐theoretical measures at each sparsity level were also calculated for statistical analysis (details in Table S3).

### 2.7 Statistical analysis

#### 2.7.1 Comparison of demographic and clinical variables

The demographic characteristics of the three groups were compared using the SPSS software, version 16.0, and the univariate one‐way analysis of variance (ANOVA) followed by the posthoc least‐significant difference tests between each pair of groups. The continuous variables of the clinical data were compared between the PKD‐M and PKD‐N groups using the independent‐sample *t* test and the categorical variables using the Chi‐square test.

#### 2.7.2 Comparison of network metrics

The structural connectome characteristics of the three groups were compared using a permutation analysis of linear models (PALM) (https://fsl.fmrib.ox.ac.uk/fsl/fslwiki/PALM), a tool implemented in FSL which allows inference using permutation methods for complex general linear models (Winkler, Ridgway, Webster, Smith, & Nichols, 2014). Nonparametric permutation tests with a design model of one‐way ANOVA were conducted to compare AUC values of all the global and nodal network metrics, among all three groups. The randomization was repeated 10,000 times. We used the default family‐wise error rate (FWER *q* value <0.05) correction for multiple comparisons in both global and nodal metrics. The less stringent correction method for nodal metrics, the Benjamini Hochberg false discovery rate (FDR; Genovese, Lazar, & Nichols, 2002), was also used as an exploratory analysis (see Table S4). Posthoc pairwise permutation tests were conducted for measures of significant group difference. In addition, to avoid the potential confounding effects of medication on structural connectome alterations in PKD patients, a subgroup analysis was performed on drug‐naive PKD‐M and PKD‐N patients. Last, though it is not unexpected that the patient group has relatively lower education levels than controls due to indirect effects of the disease, there could be an education effect on the white matter network. In an exploratory analysis we therefore performed statistical analysis on topological metrics with education as a covariate (see Table S5).

#### 2.7.3 Network matrix comparisons

To detect altered connectivity networks in PKD patients, we used a NBS approach (http://www.nitrc.org/projects/nbs/; Zalesky, Fornito, & Bullmore, 2010) with *F*‐statistics (one‐way ANOVA) to define a set of supra‐threshold links in which any connected components and their sizes could be determined (threshold, *T* = 3.85, *p* < .05). Post hoc analysis was performed for each patient group comparison with the control group, using *T*‐statistics (threshold, *T* = 2.1, *p* < .05). The significance of each supra‐threshold link among the connected components was estimated using a nonparametric permutation method (10,000 permutations).

#### 2.7.4 Correlation and regression analysis

Partial correlation analysis was used to assess the relationships in the patient groups between the AUC values of each topological metric for significant between‐group differences and the clinical variables including the age of onset and the disease duration using age and gender as covariates (*p* < .05, FDR corrected). Exploratory linear regression analysis was also conducted between the network metrics, which showed significant between‐group differences and age, and the regression coefficients of the three groups were compared. The statistical analysis was performed in SPSS 16.0.

## 3 RESULTS

### 3.1 Demographic and clinical characteristics

The demographic and clinical data are summarized in Table 1. Forty‐two patients with PKD were either drug‐naive (*n* = 28) or unmedicated (*n* = 14) for at least 48 hr before MRI scanning. There were no significant differences in age or gender among the three groups, although there was a significant difference in years of education: posthoc comparisons revealed a shorter education duration for the PKD‐M (*p* < .001) and PKD‐N (*p* < .001) groups compared to the HC group, respectively, but no significant differences between the patient subgroups were found (*p* = .772).

**TABLE 1:** Demographic and clinical characteristics of PKD‐M and PKD‐N patients and healthy controls

Characteristics	Healthy controls (*n* = 40)	PKD‐M (*n* = 35)	PKD‐N (*n* = 43)	*p* (*F*/*t*/*χ* ^2^)value	
				ANOVA	PKD‐M versus PKD‐N
Age (years)^a^	23.5 ± 4.2 (14–35)	25.5 ± 12.3 (11–62)	21.1 ± 6.5 (15–44)	0.061^b^ (2.87)	0.020^b^
Education (years)^a^	15.0 ± 2.8 (8–19)	11.6 ± 3.0 (5–16)	11.4 ± 3.2 (1–19)	<0.001^b^ (18.30)	0.772^b^
Gender (male/female)	30/10	27/7	36/7	0.491^b^ (0.97)	0.617^b^
Age of onset (years)	—	10.3 ± 4.1 (2–25)	12.5 ± 5.0 (5–36)	—	0.039^c^ (0.27)
Disease duration (years)^a^	—	15.3 ± 11.7 (2–43)	8.6 ± 7.0 (0.4–33)	—	0.004^b^ (15.11)
Family history (+/−)	—	19/16	2/41	—	<0.001^d^ (24.16)
Attack frequency (<10 per day/>10 per day)	—	27/8	21/22	—	0.011^d^ (6.53)
Affected side (left/right/bilateral/alternate)	—	4/7/17/7	8/8/13/14	—	0.323^d^ (3.48)
Phenotypes (dystonia/chorea)	—	25/10	35/8	—	0.299^d^ (1.08)
Previously‐treated/medication‐naive	—	21/14	29/14	—	0.496^d^ (0.46)
Medication (OXC/CBZ/TPM)	—	7/14/0	11/17/1	—	0.650^d^ (0.92)
Treatment time (years)^a^ ^,^ ^e^	—	2.4 ± 4.6 (0–18.1)	2.7 ± 4.4 (0–19.3)	—	0.824^c^ (0.001)

*Note:* Data are mean ± standard deviation (range in parentheses) unless otherwise indicated.

Abbreviations: ANOVA, analysis of variance; CBZ, carbamazepine; OXC, oxcarbazepine; PKD‐M/PKD‐N, paroxysmal kinesigenic dyskinesia patients with/without PRRT2 mutations; TPM, topiramate.

^a^ Age, years of education, disease duration and treatment time are as defined at the time of MRI scanning.

^b^ *p* value calculated by independent‐sample *t* test, threshold set at *p* < .05.

^c^ *p* value calculated by univariate 1‐way analysis of variance (ANOVA) followed by least‐significant differences (LSD) posthoc test, the threshold set at *p* < .05.

^d^ *p* value calculated by Chi‐squared test, threshold set at *p* < .05.

^e^ Treatment time only for medication‐treated patients.

The PKD‐M group had earlier age of onset, longer disease duration, and more frequent family history (*p* < .05) than the PKD‐N group. There was no significant difference between the PKD‐M and PKD‐N groups in clinical phenotypes, proportion of bilateral symptoms, types of medication, or treatment time.

### 3.2 Global topological alterations of the white matter structural networks

**FIGURE 1:**
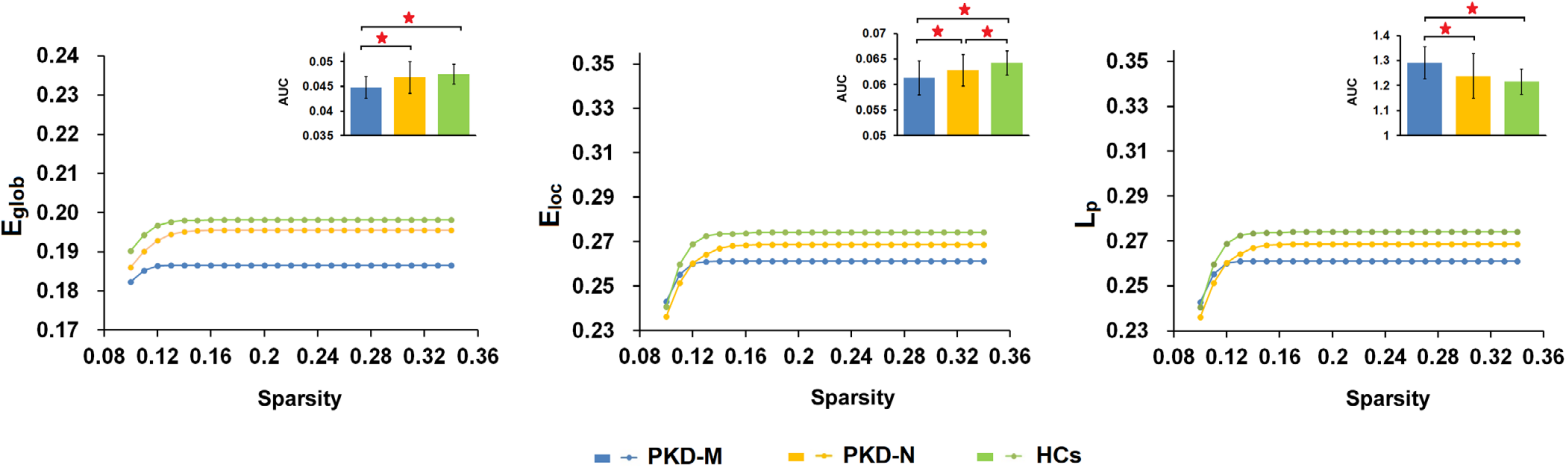
Altered global topological properties of brain white matter structural networks in PKD‐M patients, PKD‐N patients and healthy controls. The global efficiency (*E*_glob_; *p* = .0001), local efficiency (*E*_loc_; *p* = .0003), and characteristic path length (*L*_p_; *p* = .0001) significantly differ among the three groups. The figure shows *E*_glob_, *E*_loc_, and *L*_p_ of the white matter structural networks of PKD‐M patients, PKD‐N patients and healthy controls at each cost threshold (0.1–0.34, step = 0.01). Abbreviations: PKD‐M/PKD‐N = paroxysmal kinesigenic dyskinesia patients with/without *PRRT2* mutations; HCs = healthy controls. *Significant difference of topological metrics between groups at *p* < .05 with FWER correction

Both PKD and HC groups exhibited small‐world properties of the white matter structural network architecture with *γ* >1 and *λ* ≈1 (Figure S2). However, there were significant differences in specific network organization characteristics including *E*_loc_, *E*_glob_, and *L*_p_ among the three groups (*p* < .05, FWER corrected). The group difference in *E*_loc_, *E*_glob_, and *L*_p_ remained significant when only drug‐naive PKD patients and HCs were included (Table S6). No significant group differences were found in *C*_p_, *γ*, *λ*, and *σ*. Pairwise comparisons revealed significantly greater abnormalities in global network properties, with reduced E_loc_, E_glob_, and increased *L*_p_ in PKD‐M versus PKD‐N patients. (Figure 1 and Table 2).

**TABLE 2:** Brain topological metrics, showing differences among the PKD‐M and PKD‐N patient groups and healthy controls

Measurements	PKD‐M mean ± SD	PKD‐N mean ± SD	Controls mean ± SD	ANOVA *p* (*F*) values	Post hoc *p* (*t*) value		
					PKD‐M versus PKD‐N	PKD‐M versus HC	PKD‐N vs HC
*Global*							
*E*_glob_	0.0447 ± 0.0022	0.0468 ± 0.0032	0.0475 ± 0.0020	.0001^b^ (11.265)	.0002^a^ (−3.506)	.0002^a^ (−4.590)	.1182
*E*_loc_	0.0613 ± 0.0034	0.0628 ± 0.0031	0.0642 ± 0.0024	.0003^b^ (8.969)	.0164^a^ (−2.179)	.0001^a^ (−4.233)	.0154^a^ (−2.202)
*L*_p_	1.2905 ± 0.0642	1.2372 ± 0.0901	1.2156 ± 0.0509	.0001^b^ (1.803)	.0002^a^ (3.288)	.0001^a^ (4.543)	.0841
*Nodal efficiency*							
L inferior frontal gyrus	0.0428 ± 0.0040	0.0445 ± 0.0046	0.0469 ± 0.0049	.0008^b^ (7.925)	.0508	.0003^a^ (−3.935)	.0080^a^ (−2.462)
L fusiform	0.0398 ± 0.0041	0.0437 ± 0.0056	0.0445 ± 0.0052	.0002^b^ (9.036)	.0010^a^ (−3.345)	.0003^a^ (−4.018)	.2249
L thalamus	0.0526 ± 0.0046	0.0549 ± 0.0055	0.0572 ± 0.0038	.0001^b^ (8.980)	.0178^a^ (−2.155)	.0001^a^ (−4.235)	.0121^a^ (−2.228)
L middle temporal gyrus	0.0471 ± 0.0046	0.0500 ± 0.0049	0.0518 ± 0.0054	.0008^b^ (8.310)	.0057^a^ (−2.576)	.0002^a^ (−4.052)	.0560
R middle temporal gyrus	0.0450 ± 0.0044	0.0483 ± 0.0053	0.0490 ± 0.0041	.0007^b^ (8.063)	.0007^a^ (−3.154)	.0004^a^ (−3.799)	.2314

Abbreviations: ANOVA, analysis of variance; *E*_glob_, global efficiency; *E*_loc_, local efficiency; L, left; *L*_p_, characteristic path length; PKD‐M/PKD‐N, paroxysmal kinesigenic dyskinesia patients with/without PRRT2 mutations; R, right; SD, standard deviation.

^a^ Significant difference between groups at *p* < .05.

^b^ Significant difference between groups at *p* < .05 after family‐wise error rate (FWER) correction. *p* values are presented before FWER correction.

### 3.3 Nodal topological alterations of the white matter structural networks

**FIGURE 2:**
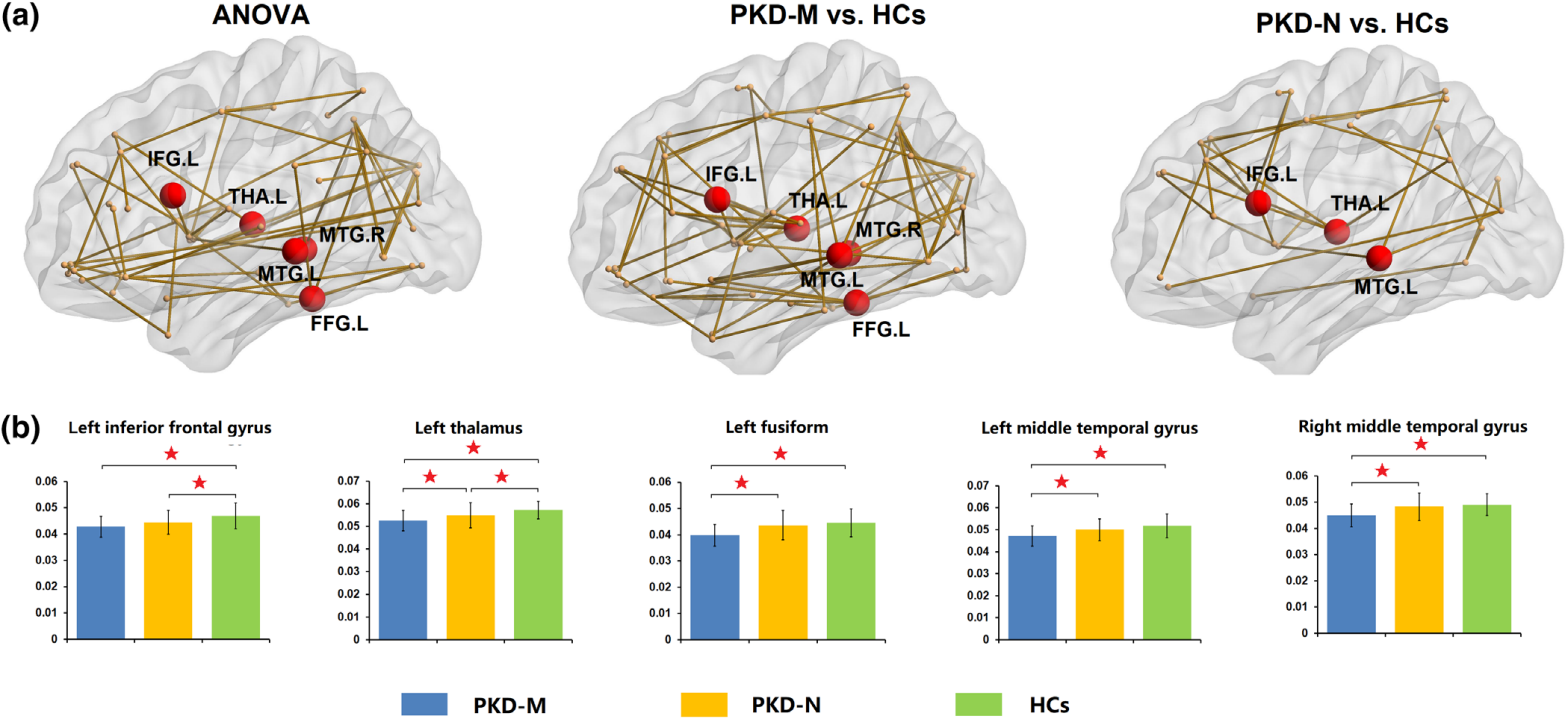
(a) Schematic of the white matter structural networks with significant group differences, between PKD‐M and HC, and between PKD‐N and HC, respectively. Note that the left connected network with F‐statistic (one‐way ANOVA, *T* = 3.85, *p* < .05) contains 55 nodes and 60 connections; the middle connected network with a *T*‐statistic (*T* = 2.1, *p* < .05) contains 69 nodes and 82 connections; and the right connected network with a *T*‐statistic (*T* = 2.1, *p* < .05) contains 32 nodes and 35 connections. The network primarily involves frontal, temporal, and occipital lobes and some subcortical regions. All the connections were decreased in patient subgroups compared with HCs. (b) Regions with altered nodal efficiency in PKD‐M patients, PKD‐N patients, and HCs. For each node, the bar and error bar represent the mean value and SD, respectively, of the nodal efficiency in each group. Abbreviations: ANOVA, analysis of variance; HCs, healthy controls, PKD‐M/PKD‐N, paroxysmal kinesigenic dyskinesia patients with/without *PRRT2* mutations, standard deviations = (SD). *Significant difference of topological metrics between groups at *p* < .05 with FWER correction

Nodal metrics for the three participant groups differed significantly in nodal efficiency in the left inferior frontal gyrus (IFG), left thalamus, left fusiform, and bilateral middle temporal gyrus (MTG; *p* < .05, FWER corrected). Posthoc tests showed that both patient groups had significantly lower nodal efficiency on the left IFG and thalamus compared to those of the HC group, whereas only the PKD‐M group showed decreased nodal efficiency on the left fusiform and bilateral MTG (Table 2, Figure 2b). Direct comparison between PKD‐M and PKD‐N patient groups revealed significantly lower nodal efficiency on the left fusiform and bilateral MTG in PKD‐M.

### 3.4 White matter structural connectivity characteristics

The NBS analysis identified a significant network of 55 nodes and 60 connections that differed between groups. These nodes were primarily located in the frontal, temporal and occipital lobes, and also in some subcortical regions (Figure 2a). Detailed information is provided in Table S7. Post hoc tests revealed that all the connections in this network were decreased in both patient subgroups compared with the HC group. However, the decreased pattern was more pronounced in PKD‐M.

### 3.5 Relationships between topological metrics and clinical variables

In each patient, we examined the relationship between the age of onset, disease duration and each network parameter, which showed significant differences among the three groups. However, no significant associations were observed in the PKD‐M or PKD‐N group. In exploratory regression analysis, the altered nodal efficiency in the left thalamus (*β* = 0.337, *p* = .027) and left IFG (*β* = 0.302, *p* = .049) showed significant age‐related increase in the PKD‐N group; also, the age‐related trends of the left thalamus were significantly different among the three groups (see Table S8 and Figure S3); In the PKD‐M group there were no age‐related changes of altered network metrics.

## 4 DISCUSSION

This is the first neuroimaging study to examine the white matter structural connectome in PKD in relation to *PRRT2* modulation. Our findings suggest general disruption of white matter structural networks (decreased *E*_loc_) in both patient groups, with the functional implication of decreased ability for segregation. More specifically, global integration ability was decreased only in the PKD‐M group (decreased *E*_glob_ and increased *L*_p_), suggesting that *PRRT2* mutations adversely affected the brain structural networks in PKD patients. Regarding the nodal and connectivity characteristics, both PKD groups showed decreased nodal efficiency in the left IFG and thalamus; however, the alterations were more pronounced in PKD‐M patients. These findings advance our understanding of the neural mechanisms of PKD itself and *PRRT2* mutations, and further suggest the potential utility of a connectome‐based approach to understand and diagnose the disease subtypes.

### 4.1 Global topological alterations in PKD‐M and PKD‐N patients

A small‐world network pattern reflects an optimal balance between network segregation (measured by *C*_p_, *γ*, and *E*_loc_) and network integration (measured by *L*_p_, *λ*, and *E*_glob_) abilities (Rubinov & Sporns, 2010; Suo et al., 2018). Although it retains overall small‐world architecture, the structural connectome in PKD‐N patients is prone to a network breakdown (reflected by decreased *E*_loc_) implying weaker local segregation ability. Conversely, decreased *E*_glob_ and increased *L*_p_ were also found in the PKD‐M group, indicating a *PRRT2* mutation‐related impact on human neurophysiology. Loss‐of‐function mutations in *PRRT2* could lead to synaptic dysfunction; our observation is in accordance with the results of a magnetoencephalographic study which showed that *PRRT2*‐related PKD patients had greater reduction in peak gamma frequency than non‐*PRRT* PKD patients (Z. R. Liu, Miao, Yu, Ding, & Liao, 2016). Consistent with this, the *PRRT2* knockout mouse shows impaired functional stability of neuronal networks resulting from short‐term plasticity changes, rendering it more susceptible to paroxysmal events triggered by external stimuli (Michetti et al., 2017). In the PKD‐M group, the white matter structural networks shifted toward “weaker small‐worldness,” with reduction in both global integration and local segregation. This suggests that mutations in *PRRT2*, a network stability gene (Michetti et al., 2017), render the brain structural networks of PKD patients less efficient at both local and global scales.

### 4.2 Nodal topological alterations in PKD‐M and PKD‐N patients

In addition to these global topological abnormalities, we found topological alterations in several brain regions related to PKD and *PRRT2* mutations. Our results regarding the thalamus are consistent with previous studies reporting thalamic structural and functional abnormalities in PKD patients (J. H. Kim et al., 2015; Long et al., 2017; Shirane et al., 2001; Zhou, Chen, Gong, et al., 2010). The thalamus is a major “gateway” sensory relay station connecting the cortical and subcortical regions (Long et al., 2017; Theyel, Llano, & Sherman, 2010). The striatum (putamen and caudate) receives somatosensory intracortical inhibitory impulses and sends inhibitory projections to the thalamus; the thalamus in turn, projects excitatory fibers to the motor and supplementary motor cortex and finally to the spinal motor neurons which stimulate muscle contractions (Breakefield et al., 2008; J. H. Kim et al., 2015; Peterson, Sejnowski, & Poizner, 2010). Thus, impaired inhibitory control of the basal ganglia and thalamus could result in overactivity of the thalamo‐cortical circuit, leading to hyperkinetic symptoms such as chorea, dystonia, and ballism.

The decreased efficiency in the left IFG in PKD patients compared to HCs is consistent with a previous study reporting decreased gray matter volume in IFG in PKD patients (H. F. Li, Yang, et al., 2019), and a study comparing PKD patients with dystonia, PKD patients with chorea and HCs, which found significant differences of regional homogeneity (ReHo) in the IFG (Z. R. Liu et al., 2016). The IFG (specially the opercular part as detected in this study) is centrally involved in motor inhibition, and connected with the presupplementary motor area and the subthalamic nucleus (Neubert, Mars, Buch, Olivier, & Rushworth, 2010; Swann et al., 2012). Our findings of decreased nodal efficiency in these two regions, the thalamus and IFG, add to the evidence that white matter pathways within the basal ganglia‐thalamo‐cortical circuit are affected in PKD.

Though both groups showed decreased nodal efficiency in the left IFG and thalamus, the changes were more pronounced in PKD‐M patients. Directly comparing PKD‐M with PKD‐N groups, we found significantly decreased nodal efficiency in the left thalamus in PKD‐M. In addition, the exploratory regression analysis revealed a significant age‐related increase in nodal efficiency in the left thalamus in the PKD‐N group, but no similar age‐related trends in PKD‐M. Previous studies demonstrated age‐related trajectories of nodal efficiency in the prefrontal cortex and basal ganglia that increased linearly during development (Collin & van den Heuvel, 2013; Zhao et al., 2015). Our results suggest that connectivity in the left thalamus may decline or fail to develop in PKD‐M, but not in PKD‐N patients. While the mechanism is uncertain, it is possible that *PRRT2* affects the neural development in ways that bring consequences in the white matter structural networks in PKD.

In addition, the PKD‐M group had more regional disturbances, such as decreased nodal efficiency in the left fusiform and bilateral MTG compared to the PKD‐N group and the HC group. This is in line with a previous hypothesis that the pathophysiology of PKD cannot be explained by the lesions only in the basal ganglia‐thalamo‐cortical circuit, but depends also on abnormalities in other associated areas (Y. D. Kim et al., 2011; Ren et al., 2015). A single photon emission computed tomography study found perfusion changes in the left temporal cortex in PKD patients compared to controls (Y. D. Kim et al., 2011). Interestingly, a clinical study reported that PKD of epileptic origin was abolished by temporal lobectomy (Aybek, Rossetti, Maeder‐Ingvar, & Vingerhoets, 2012). Decreased nodal centrality in the MTG has also been reported in epilepsy (M. Liu, Chen, Beaulieu, & Gross, 2014), which may partly explain both the overlap of symptoms between PKD and epilepsy and the therapeutic effect of antiepileptic agents in PKD. It is also noteworthy that *PRRT2* mutations were identified in both PKD patients and epilepsy patients (Okumura et al., 2019). We suggest that the nodal alterations in the temporal lobe observed only in PKD‐M patients may be induced by *PRRT2* mutations. Previous studies of the *PRRT2* expression profile have reported high levels of *PRRT2* mRNA in many tissues of the nervous system, including the cerebral cortex, hippocampus, and basal ganglia (W. J. Chen et al., 2011; Su et al., 2004). Specifically, *PRRT2* is found in the small translucent vesicles localized to the axon terminals and presynaptic structures (Tan et al., 2018). Truncating mutations within the *PRRT2* gene resulted in an altered subcellular location of the PRRT2 protein, which regulates the key properties of ion channels related to PKD symptoms. As ion‐channel blockers such as carbamazepine are highly effective (Bhatia, Griggs, & Ptacek, 2000; Celesia, 2001), it has been proposed that PKD might be a channelopathy. Consistent with this proposal, we also found white matter pathway abnormalities in the temporal lobe. Taken together, our results support the idea that the basal ganglia‐thalamo‐cortical circuit abnormalities may be a neural marker of PKD directly linked to the disease process in both PKD‐M and PKD‐N groups, whereas the more pronounced diminished nodal centralities in the temporal cortex may be due to secondary plasticity changes related to the functional effects of *PRRT2* mutation.

### 4.3 Limitations

Our study has some limitations. Some patients were on routine medication, which could interfere to the alterations of the structural connectome; however, the type and treatment time of the drugs did not differ between the two patient groups. Second, we used an AAL template separating the brain into 90 regions, and this may introduce potential bias due to the regions' inhomogeneity with relatively small numbers of nodes created in the whole brain; differences in template type may cause considerable variations in the graph‐based theoretical parameters. For example, a previous functional study investigated the impact of different parcellation choice (AAL vs. Dosenbach template) on comparing graph properties in patients with anorexia nervosa and HCs, and observed robust patient‐specific global network properties but with variation in nodal locations (Lord et al., 2016). Thus, though there is no gold standard for template choice and its impact on detecting disease effects remains unclear, the effect of the numbers of nodes in brain network should not be dismissed. Future study using different parcellation schemes to explore the graph properties in PKD patients could be performed. Third, patients with *PRRT2*‐negative mutations might have other potential causative genes, which should also be considered in future studies (Tian et al., 2018). Fourth, we used deterministic fiber tractography that might reduce sensitivity as the tracking procedure ceases at the regions with fiber crossing. Future studies with advanced methods are needed to improve the accuracy of tractography. Finally, we have not explored alteration of the white‐matter functional connectome in PKD, which is a novel perspective and could be detected using blood oxygen level‐dependent‐functional MRI to estimate white matter function (Ji et al., 2019; J. Li, Biswal, et al., 2019).

## 5 CONCLUSION

These alterations of topological organization in the brain structural networks provide some insights into the interacting pathophysiological mechanisms of PKD and the effects of the PRRT2 mutation revealing the general white matter alterations of basal ganglia‐thalamo‐cortical circuit related to PKD, with or without *PRRT2* mutation, our results provide insight into the white matter structural network disturbances related to specific features of disease phenomenology. The findings of weaker small‐worldness and regional disturbance in the temporal regions, along with more severe clinical manifestations (earlier age of onset, longer duration of attacks) in the PKD‐M group specifically, point to the adverse effects of *PRRT2* mutation on brain structural connectome.

## CONFLICT OF INTERESTS

All authors declare no competing interests.

## DATA AVAILABILITY STATEMENT

The data that support the findings of this study are available from the corresponding author upon reasonable request. And the data and code sharing adopted by the authors comply with the requirements of the funding institute, and comply with institutional ethics approval.

## Supporting information


**Appendix** **S1:** Supporting information
