# Supplementary material for: Brain structural connectome in relation to PRRT2 mutations in paroxysmal kinesigenic dyskinesia
Source: Hum Brain Mapp. 2020 Jun 27;41(14):3855–66. doi: 10.1002/hbm.25091 (PMC7469858; doi:10.1002/hbm.25091)
Supplement: Supplementary file 1 — Appendix S1: Supporting information [file HBM-41-3855-s001.doc]

**Supplementary Material**

**Methods and Materials**

**Quality control procedures for MRI images**

A water phantom was used as daily quality assurance protocol to establish the stability of the MRI system. For all the raw DTI datasets, first we carefully checked by visual inspection to exclude those with conspicuous head motion and signal dropout. Then we calculated the head motion using on-line code (<https://thewinnower.com/papers/3525-a-guide-to-quantifying-head-motion-in-dti-studies>). Participants with head movements of displacement or translation at x, y, z direction >2 mm or rotation at x, y, z direction more than 2° during MRI scans would be excluded. All subjects with data reported in the paper had head motion less than the exclusion criteria. Details of the head motion parameters were presented in Table S1.

**Results**

**Table S1: Comparison of head movement parameters among the PKD-M and PKD-N patients and healthy controls**

| Measurements | PKD-M  Mean ± SD | PKD-N  Mean ± SD | HC  Mean ± SD | ANOVA  *p* (F) values | Post hoc *p* value | | |
| --- | --- | --- | --- | --- | --- | --- | --- |
|  |  |  |  |  | PKD-M vs PKD-N | PKD-M vs HC | PKD-N vs HC |
| Relative displacement | 0.3769 ± 0.2049 | 0.4712 ± 0.3063 | 0.4925 ± 0.2892 | 0.161 (1.856) | 0.133 | 0.071 | 0.724 |
| Rotation at x direction | -0.0008 ± 0.0112 | -0.0008 ± 0.0086 | -0.0052 ± 0.0360 | 0.600 (0.513) | 0.993 | 0.394 | 0.374 |
| Rotation at y direction s | -0.0001 ± 0.0069 | 0.0002 ± 0.0068 | 0.0104 ± 0.0633 | 0.367 (1.010) | 0.968 | 0.227 | 0.218 |
| Rotation at z direction | 0.0005 ± 0.0075 | -0.0001 ± 0.0051 | -0.0133 ± 0.0758 | 0.302 (1.211) | 0.952 | 0.183 | 0.180 |
| Translation at x direction | 0.0109 ± 0.4677 | 0.0331 ± 0.3049 | -0.0743 ± 0.3207 | 0.380(0.977) | 0.790 | 0.316 | 0.183 |
| Translation at y direction | 0.6370 ± 0.2572 | 0.5897 ± 0.1714 | 0.6387 ± 0.1614 | 0.446 (0.814) | 0.295 | 0.971 | 0.261 |
| Translation at z direction | 0.4138 ± 0.5185 | 0.5528 ± 0.6936 | 0.4817 ± 0.4712 | 0.569 (0.567) | 0.290 | 0.611 | 0.574 |

Abbreviations: HC healthy controls, PKD-M/PKD-N paroxysmal kinesigenic dyskinesia patients with/without PRRT2 mutations, ANOVA analysis of variance, SD standard deviation.

**Table S2. Comparison of topological metrics among PKD-M and PKD-N patients and healthy controls using weighted FN matrix, weighted FN*FA matrix, binary FA matrix, binary FN matrix, and binary FN*FA matrix**

| Measurements | ANOVA *p* (F) values | | | | |
| --- | --- | --- | --- | --- | --- |
|  | FA_binary | FN_weighted | FN_binary | FN*FA_weighted | FN*FA_binary |
| **Global** |  |  |  |  |  |
| E_glob_ | 0.0244^*^ (3.890) | 0.6122 | 0.0166^*^ (4.311) | 0.0173^*^ (4.160) | 0.1652 |
| E_loc_ | 0.6844 | 0.8922 | 0.2996 | 0.0364^*^ (3.335) | 0.9954 |
| L_p_ | 0.0244^*^ (3.902) | 0.2294 | 0.0159^*^ (4.350) | 0.0623 | 0.1880 |
| **Nodal efficiency** |  |  |  |  |  |
| L inferior frontal gyrus | 0.1225 | 0.8501 | 0.1171 | 0.2373 | 0.4775 |
| L fusiform | 0.1387 | 0.4678 | 0.1163 | 0.0689 | 0.6423 |
| L thalamus | 0.0232^*^ (3.826) | 0.7601 | 0.0261^*^ (3.724) | 0.1040 | 0.6078 |
| L middle temporal gyrus | 0.0390 | 0.3913 | 0.0367^*^ (3.447) | 0.1793 | 0.0834 |
| R middle temporal gyrus | 0.1114 | 0.5721 | 0.0861 | 0.0424^*^ (3.186) | 0.4801 |

Abbreviations: PKD-M/PKD-N paroxysmal kinesigenic dyskinesia patients with/without PRRT2 mutations, L left, R right.

^*^ Significant difference of topological metrics between groups at *p* < 0.05.

**Table S3. Comparison of topological metrics among PKD-M and PKD-N patients and healthy controls at each spar**sity level

| Measurements | ANOVA *p* (F) values | | | | |  |
| --- | --- | --- | --- | --- | --- | --- |
|  | Sparsity 0.1 | Sparsity 0.11 | Sparsity 0.12 | Sparsity 0.13 | Sparsity 0.14 | Sparsity 0.34 |
| **Global** |  |  |  |  |  |  |
| E_glob_ | 0.0001^#^ (11.593) | 0.0001^#^ (11.593) | 0.0002^#^ (11.398) | 0.0001^#^ (11.577) | 0.0001^#^ (11.390) | 0.0001^#^ (11.143) |
| E_loc_ | 0.1077 | 0.0077^#^ (5.074) | 0.0013^#^ (7.283) | 0.0005^#^ (8.807) | 0.0005^#^ (8.568) | 0.0002^#^ (8.993) |
| L_p_ | 0.0001^#^ (10.284) | 0.0001^#^ (10.651) | 0.0002^#^ (10.628) | 0.0001^#^ (10.958) | 0.0001^#^ (10.865) | 0.0001^#^ (10.753) |
| **Nodal efficiency** |  |  |  |  |  |  |
| L inferior frontal gyrus | 0.0013^*^ (7.051) | 0.0011^*^ (7.396) | 0.0007^#^(7.930) | 0.0007^#^(8.059) | 0.0008^#^(7.972) | 0.0008^#^(7.956) |
| L fusiform | 0.0022^*^ (6.471) | 0.0006^#^(8.134) | 0.0003^#^(8.863) | 0.0002^#^(9.201) | 0.0002^#^(9.146) | 0.0002^#^(9.125) |
| L thalamus | 0.0004^#^(8.279) | 0.0001^#^ (8.889) | 0.0001^#^ (8.821) | 0.0001^#^ (9.019) | 0.0001^#^ (9.030) | 0.0001^#^ (8.999) |
| L middle temporal gyrus | 0.0019^*^ (7.069) | 0.0001^#^ (7.861) | 0.0011^#^ (8.033) | 0.0008^#^ (8.353) | 0.0007^#^ (8.358) | 0.0006^#^ (8.358) |
| R middle temporal gyrus | 0.0037^*^ (5.872) | 0.0022^*^ (6.416) | 0.0007^*^ (7.512) | 0.0007^#^ (7.997) | 0.0007^#^ (8.086) | 0.0006^#^ (8.221) |

Abbreviations: PKD-M/PKD-N paroxysmal kinesigenic dyskinesia patients with/without PRRT2 mutations, L left, R right

^*^ Significant difference of topological metrics between groups at *p* < 0.05.

^#^ Significant difference of topological metrics between groups at *p* < 0.05 after family-wise error rate (FWER) correction (n = 90). P values are presented before FWER correction.

**Table S4. Regions showing altered nodal centralities among the PKD-M and PKD-N patients and healthy controls at *p* < 0.05 with Benjamini Hochberg False Discovery Rate (FDR)** correction.

| Brain Regions |  | Mean ± SD |  | ANOVA  *p* (F) values | ANOVA  FDR q values |  |
| --- | --- | --- | --- | --- | --- | --- |
|  | PKD-M | PKD-N | Healthy Controls |  |  |  |
| **Nodal efficiency** |  |  |  |  |  |  |
| Left Precentral gyrus | 0.0502 ± 0.0047 | 0.0517 ± 0.0040 | 0.026 ± 0.0024 | 0.016 (4.177) | 0.0411 |  |
| Left superior frontal gyrus, dorsolateral | 0.0472 ± 0.0041 | 0.0469 ± 0.0040 | 0.0499 ± 0.0050 | 0.0038 (5.779) | 0.0167 |  |
| Right middle frontal gyrus | 0.0431 ± 0.0039 | 0.0444 ± 0.0056 | 0.0470 ± 0.0040 | 0.0009 (7.081) | 0.009 |  |
| Left inferior frontal gyrus, opercular part | 0.0428 ± 0.0040 | 0.0445 ± 0.0046 | 0.0469 ± 0.0049 | 0.0008 (7.925) | 0.009 |  |
| Right inferior frontal gyrus, opercular part | 0.0437 ± 0.0046 | 0.0438 ± 0.0056 | 0.0474 ± 0.0051 | 0.0021 (6.652) | 0.0135 |  |
| Right inferior frontal gyrus, triangular part | 0.0454 ± 0.0053 | 0.0483 ± 0.0069 | 0.0504 ± 0.0049 | 0.0019 (6.935) | 0.0132 |  |
| Right inferior frontal gyrus, orbital part | 0.0456 ± 0.0048 | 0.0490 ± 0.0065 | 0.0490 ± 0.0054 | 0.0133 (4.408) | 0.0386 |  |
| Right rolandic operculum | 0.0389 ± 0.0038 | 0.0414 ± 0.0049 | 0.0423 ± 0.0040 | 0.0037 (5.998) | 0.0167 |  |
| Right supplementary motor area | 0.0484 ± 0.0045 | 0.0499 ± 0.0048 | 0.0518 ± 0.0038 | 0.0038 (5.773) | 0.0167 |  |
| Left olfactory cortex | 0.0394 ± 0.0048 | 0.0434 ± 0.0061 | 0.0437 ± 0.0053 | 0.0012 (7.311) | 0.0098 |  |
| Right superior frontal gyrus, medial | 0.0449 ± 0.0045 | 0.0474 ± 0.0041 | 0.0487 ± 0.0043 | 0.001 (7.324) | 0.009 |  |
| Left superior frontal gyrus, medial orbital | 0.0439 ± 0.0049 | 0.0459 ± 0.0058 | 0.0473 ± 0.0043 | 0.0155 (4.152) | 0.0411 |  |
| Right superior frontal gyrus, medial orbital | 0.0430 ± 0.0044 | 0.0469 ± 0.0066 | 0.0463 ± 0.0042 | 0.0039 (5.963) | 0.0167 |  |
| Left gyrus rectus | 0.0433 ± 0.0056 | 0.0456 ± 0.0061 | 0.0471 ± 0.0043 | 0.0105 (4.709) | 0.0315 |  |
| Right insula | 0.0417 ± 0.0050 | 0.0452 ± 0.0060 | 0.0432 ± 0.0050 | 0.0159 (4.298) | 0.0411 |  |
| Right median cingulate gyrus | 0.0465 ± 0.0039 | 0.0480 ± 0.0044 | 0.0490 ± 0.0023 | 0.0166 (4.268) | 0.0415 |  |
| Left posterior cingulate gyrus | 0.0442 ± 0.0041 | 0.0469 ± 0.0049 | 0.0475 ± 0.0037 | 0.0026 (6.186) | 0.0156 |  |
| Left hippocampus | 0.0467 ± 0.0042 | 0.0495 ± 0.0053 | 0.0498 ± 0.0048 | 0.0157 (4.419) | 0.0411 |  |
| Right hippocampus | 0.0485 ± 0.0040 | 0.0525 ± 0.0062 | 0.0509 ± 0.0045 | 0.0032 (6.056) | 0.0167 |  |
| Left parahippocampal gyrus | 0.0379 ± 0.0034 | 0.0405 ± 0.0040 | 0.0412 ± 0.0041 | 0.0005 (7.718) | 0.009 |  |
| Left calcarine fissure and surrounding cortex | 0.0528 ± 0.0058 | 0.0561 ± 0.0057 | 0.0560 ± 0.0054 | 0.0202 (4.015) | 0.0466 |  |
| Right calcarine fissure | 0.0531 ± 0.0048 | 0.0555 ± 0.0055 | 0.0564 ± 0.0040 | 0.008 (4.690) | 0.0257 |  |
| Right cuneus | 0.0521 ± 0.0046 | 0.0550 ± 0.0057 | 0.0560 ± 0.0055 | 0.0071 (5.315) | 0.0237 |  |
| Left lingual gyrus | 0.0444 ± 0.0047 | 0.0473 ± 0.0054 | 0.0490 ± 0.0050 | 0.0006 (7.632) | 0.009 |  |
| Right lingual gyrus | 0.0442 ± 0.0048 | 0.0470 ±0.0051 | 0.0483 ± 0.0057 | 0.0043 (6.062) | 0.0168 |  |
| Right superior occipital gyrus | 0.0547 ± 0.0047 | 0.0559 ± 0.0051 | 0.0582 ± 0.0043 | 0.0043 (5.292) | 0.0168 |  |
| Left fusiform gyrus | 0.0398 ± 0.0041 | 0.0437 ± 0.0056 | 0.0445 ± 0.0052 | 0.0002 (9.036) | 0.0009 |  |
| Left superior parietal gyrus | 0.0471 ± 0.0048 | 0.0502 ± 0.0057 | 0.0509 ± 0.0055 | 0.0049 (5.332) | 0.017 |  |
| Left angular gyrus | 0.0405 ± 0.0040 | 0.0435 ± 0.0065 | 0.0443 ± 0.0046 | 0.0046 (5.426) | 0.017 |  |
| Right angular gyrus | 0.0435 ± 0.0050 | 0.0477 ± 0.0087 | 0.0469 ± 0.0057 | 0.0191 (4.067) | 0.0465 |  |
| Left precuneus | 0.0562 ± 0.0052 | 0.0587 ± 0.0062 | 0.0600 ±0.0042 | 0.0083 (4.954) | 0.0258 |  |
| Left caudate nucleus | 0.0477 ± 0.0043 | 0.0494 ± 0.0062 | 0.0516 ± 0.0040 | 0.003 (6.031) | 0.0167 |  |
| Right caudate nucleus | 0.0475 ± 0.0041 | 0.0509 ± 0.0061 | 0.0512 ± 0.0035 | 0.0016 (6.753) | 0.012 |  |
| Left thalamus | 0.0526 ± 0.0046 | 0.0549 ± 0.0055 | 0.0572 ± 0.0038 | 0.0001 (8.98) | 0.009 |  |
| Right superior temporal gyrus | 0.0421 ± 0.0050 | 0.0458 ± 0.0054 | 0.0457 ± 0.0060 | 0.0049 (5.431) | 0.017 |  |
| Left temporal pole: superior temporal gyrus | 0.0415 ± 0.0049 | 0.0444 ± 0.0049 | 0.0446 ± 0.0054 | 0.0199 (4.155) | 0.0466 |  |
| Left middle temporal gyrus | 0.0471 ± 0.0046 | 0.0500 ± 0.0049 | 0.0518 ± 0.0054 | 0.0008 (8.310) | 0.009 |  |
| Right middle temporal gyrus | 0.0450 ± 0.0044 | 0.0483 ± 0.0053 | 0.0490 ± 0.0041 | 0.0007 (8.063) | 0.009 |  |
| Left temporal pole: middle temporal gyrus | 0.0385 ± 0.0106 | 0.0431 ± 0.0055 | 0.0446 ± 0.0046 | 0.0004 (7.161) | 0.009 |  |

**Table S5. Comparison of topological metrics among PKD-M and PKD-N patients and healthy controls with and without education as covariate and education-related effects on topological metrics**

Abbreviations: *L_p_* characteristic path length, *E_glob_* global efficiency, *E_loc_* local efficiency, HC healthy controls, PKD-M/PKD-N paroxysmal kinesigenic dyskinesia patients with/without PRRT2 mutations, *L* left, *R* right.

| Measurements | Without education  p (F) values | With education  p (F) values | Education effect  p (F) values |  |
| --- | --- | --- | --- | --- |
|
| **Global** |  |  |  |  |
| E_glob_ | 0.0001^#^ (11.265) | 0.0004^#^ (8.419) | 0.0395^*^ (1.754) |  |
| E_loc_ | 0.0003^#^ (8.969) | 0.0032^#^ (6.514) | 0.2561 (0.644) |  |
| L_p_ | 0.0001^#^ (10.803) | 0.0006^#^ (7.832) | 0.0468^*^ (1.713) |  |
| **Nodal efficiency** |  |  |  |  |
| L inferior frontal gyrus | 0.0008^#^ (7.925) | 0.0083^*^ (4.961) | 0.1457 (1.035) |  |
| L fusiform | 0.0002^#^ (9.036) | 0.0012^*^ (7.123) | 0.0826 (1.418) |  |
| L thalamus | 0.0001^#^ (8.980) | 0.0064^*^ (5.472) | 0.0639 (1.563) |  |
| L middle temporal gyrus | 0.0008^#^ (8.310) | 0.0059^*^ (5.458) | 0.0473^*^ (1.679) |  |
| R middle temporal gyrus | 0.0007^#^ (8.063) | 0.0043^*^ (5.981) | 0.0104^*^ (2.318) |  |

^*^ Significant difference of topological metrics between groups at *p* < 0.05. ^#^ Significant difference of topological metrics between groups at *p* < 0.05 after family-wise error rate (FWER) correction (n = 90). P values are presented before FWER correction.

**Table S6. Brain topological metrics showing differences among the drug-naïve PKD-M and drug-naive PKD-N patient groups and healthy controls**

| Measurements | PKD-M (n=14)  Mean ± SD | PKD-N (n=14)  Mean ± SD | HC (n=40)  Mean ± SD | ANOVA  *p* (F) values | Post hoc *p* (t) value | | |
| --- | --- | --- | --- | --- | --- | --- | --- |
|  |  |  |  |  | PKD-M vs PKD-N | PKD-M vs HC  *p* (t) value | PKD-N vs HC |
| **Global** |  |  |  |  |  |  |  |
| E_glob_ | 0.0450 ± 0.0020 | 0.0465 ± 0.0042 | 0.0475 ± 0.0020 | 0.0135^*^ (4.611) | 0.0659 | 0.0022^*^ (-3.009) | 0.8738 |
| E_loc_ | 0.0611 ± 0.0029 | 0.0621 ± 0.0041 | 0.0642 ± 0.0024 | 0.0013^#^ (7.138) | 0.1824 | 0.0010^*^ (-3.467) | 0.9870 |
| L_p_ | 1.2819 ± 0.0598 | 1.2488 ± 0.1242 | 1.2156 ± 0.0509 | 0.0141^*^ (4.516) | 0.1168 | 0.0039^*^ (2.915) | 0.0826 |
| **Nodal efficiency** |  |  |  |  |  |  |  |
| L inferior frontal gyrus | 0.0426 ± 0.0037 | 0.0428 ± 0.0049 | 0.0469 ± 0.0049 | 0.0027^*^ (6.803) | 0.4544 | 0.0020^*^ (-2.993) | 0.9969 |
| L fusiform | 0.0399 ± 0.0030 | 0.0435 ± 0.0044 | 0.0445 ± 0.0052 | 0.0087^*^ (5.054) | 0.0214^*^ (-2.040) | 0.0011^*^ (-3.177) | 0.7519 |
| L thalamus | 0.0530 ± 0.0033 | 0.0540 ± 0.0060 | 0.0572 ± 0.0038 | 0.0032^*^ (6.333) | 0.2661 | 0.0006^*^ (-3.164) | 0.9892 |
| L middle temporal gyrus | 0.0464 ± 0.0041 | 0.0518 ± 0.0045 | 0.0518 ± 0.0054 | 0.0028^*^ (6.472) | 0.0031^*^ (-2.871) | 0.0009^*^ (-3.468) | 0.4943 |
| R middle temporal gyrus | 0.0450 ± 0.0041 | 0.0496 ± 0.0061 | 0.0490 ± 0.0041 | 0.0126^*^ (4.667) | 0.0057^*^ (-2.637) | 0.0025^*^ (-2.829) | 0.3451 |

Abbreviations: *L_p_* characteristic path length, *E_glob_* global efficiency, *E_loc_* local efficiency, HC healthy controls, PKD-M/PKD-N paroxysmal kinesigenic dyskinesia patients with/without PRRT2 mutations, ANOVA analysis of variance, SD standard deviation, *L* left, *R* right.

^*^ Significant difference of topological metrics between groups at *p* < 0.05.

^#^ Significant difference of topological metrics between groups at *p* < 0.05 after family-wise error rate (FWER) correction (n = 90). P values are presented before FWER correction.

**Table S7. Connections with group difference among three groups (PKD-M and PKD-N patients and healthy controls) using the network-based statistics approach.**

| **Region 1** | **Region 2** | **F statistic** | **Region 1** | **Region 2** | **F statistic** |
| --- | --- | --- | --- | --- | --- |
| PreCG.L | MFG.L | 4.70 | FFG.L | SMG.L | 4.25 |
| SFGdor.L | MFG.L | 9.44 | PreCG.L | ANG.L | 4.28 |
| MFG.L | ORBmid.L | 4.67 | SOG.R | ANG.L | 4.68 |
| MFG.L | IFGtriang.L | 10.68 | FFG.L | ANG.L | 4.13 |
| ORBinf.R | SFGmed.R | 9.11 | SMG.L | ANG.L | 4.47 |
| ORBmid.R | ORBsupmed.R | 4.12 | CUN.R | ANG.R | 6.05 |
| ORBinf.R | ORBsupmed.R | 3.92 | ANG.L | ANG.R | 4.96 |
| ORBsupmed.L | ORBsupmed.R | 4.09 | CAL.L | PCUN.L | 7.85 |
| ORBinf.R | REC.L | 4.72 | CAL.R | PCUN.L | 6.15 |
| MFG.L | INS.L | 8.45 | LING.L | PCUN.L | 3.86 |
| IFGoperc.L | INS.L | 3.99 | LING.L | PCUN.R | 4.76 |
| ROL.L | INS.L | 4.64 | ANG.R | PCUN.R | 4.14 |
| PreCG.R | INS.R | 4.64 | SFGmed.L | CAU.L | 4.37 |
| ROL.R | INS.R | 4.64 | ORBsupmed.R | CAU.L | 5.00 |
| ORBinf.L | ACG.L | 4.96 | ORBinf.L | CAU.R | 4.88 |
| ACG.L | ACG.R | 5.28 | PCUN.R | CAU.R | 4.78 |
| ORBinf.L | HIP.R | 4.76 | ROL.R | PUT.R | 7.44 |
| ORBsupmed.R | CAL.R | 5.89 | CAU.L | PUT.R | 4.38 |
| CUN.L | LING.L | 6.61 | SOG.R | PAL.R | 4.69 |
| CAL.R | LING.R | 5.58 | SFGdor.L | THA.L | 4.27 |
| CUN.R | LING.R | 5.82 | ORBinf.L | STG.L | 4.87 |
| PCG.L | SOG.R | 4.77 | SOG.R | STG.R | 5.53 |
| ORBsup.L | MOG.L | 5.87 | IOG.L | TPOsup.L | 4.74 |
| ORBmid.L | MOG.L | 4.69 | PUT.R | MTG.L | 4.16 |
| CUN.L | MOG.L | 4.71 | PCUN.R | MTG.R | 3.90 |
| ORBinf.L | FFG.L | 4.42 | ORBsupmed.L | TPOmid.L | 4.46 |
| IOG.L | FFG.L | 5.09 | CAU.L | TPOmid.L | 3.95 |
| PreCG.L | PoCG.R | 4.41 | HIP.R | ITG.R | 5.20 |
| PreCG.L | SPG.L | 4.46 | IOG.R | ITG.R | 4.79 |
| SPG.L | IPL.R | 3.85 | CAU.R | ITG.R | 4.80 |

**Table S8. Age-related changes in brain white matter networks in the PKD-M and PKD-N patients and healthy controls**

|  | Regression for PKD-M | | Regression for PKD-N | | Regression for HCs | | Regression for group comparison | |
| --- | --- | --- | --- | --- | --- | --- | --- | --- |
| Measurements | β | *p* value | β | *p* value | β | *p* value | β | *p* value |
| **Global** |  |  |  |  |  |  |  |  |
| E_glob_ | -0.008 | 0.965 | 0.225 | 0.148 | 0.126 | 0.439 | 0.0398 | 0.275 |
| E_loc_ | -0.026 | 0.882 | 0.267 | 0.084 | 0.057 | 0.729 | 0.386 | 0.293 |
| L_p_ | 0.005 | 0.975 | -0.241 | 0.119 | -0.115 | 0.479 | -0.401 | 0.272 |
| **Nodal efficiency** |  |  |  |  |  |  |  |  |
| L inferior frontal gyrus | -0.028 | 0.874 | 0.302 | 0.049^*^ | -0.027 | 0.867 | 0.305 | 0.411 |
| L fusiform | 0.036 | 0.839 | 0.155 | 0.321 | -0.277 | 0.084 | -0.390 | 0.295 |
| L thalamus | -0.113 | 0.518 | 0.337 | 0.027^*^ | 0.176 | 0.276 | 0.757 | 0.038^*^ |
| L middle temporal gyrus | 0.079 | 0.652 | 0.005 | 0.975 | 0.072 | 0.657 | 0.028 | 0.940 |
| R middle temporal gyrus | -0.012 | 0.945 | 0.097 | 0.538 | -0.029 | 0.977 | 0.064 | 0.864 |

Abbreviations: *L_p_* characteristic path length, *E_glob_* global efficiency, *E_loc_* local efficiency, HC healthy controls, PKD-M/PKD-N paroxysmal kinesigenic dyskinesia patients with/without PRRT2 mutations, ANOVA analysis of variance, SD standard deviation, *L* left, *R* right.

^*^ Significant difference of topological metrics between groups at *p* < 0.05

Figure S1. An example of the FA matrix, FN matrix, and FA*FN matrix, respectively, in a patient with paroxysmal kinesigenic dyskinesia.


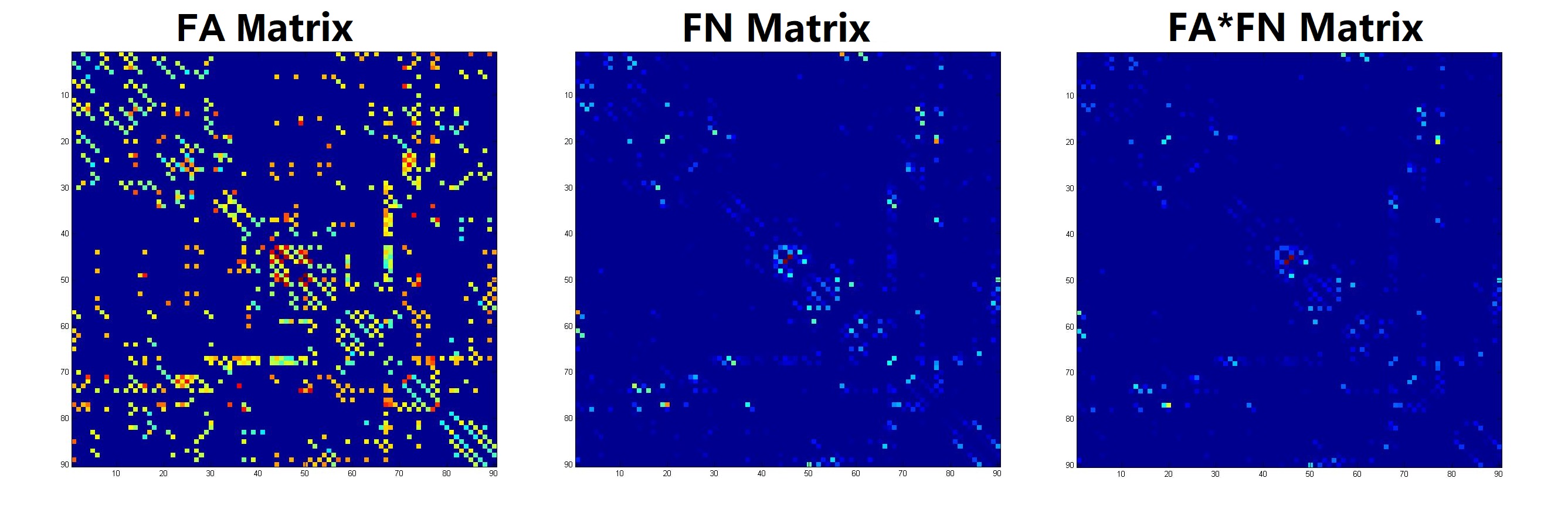


Abbreviations: FA, fractional anisotropy; FN, fiber number

Figure S2. The key small-world parameters of the white matter networks as a function of sparsity thresholds.


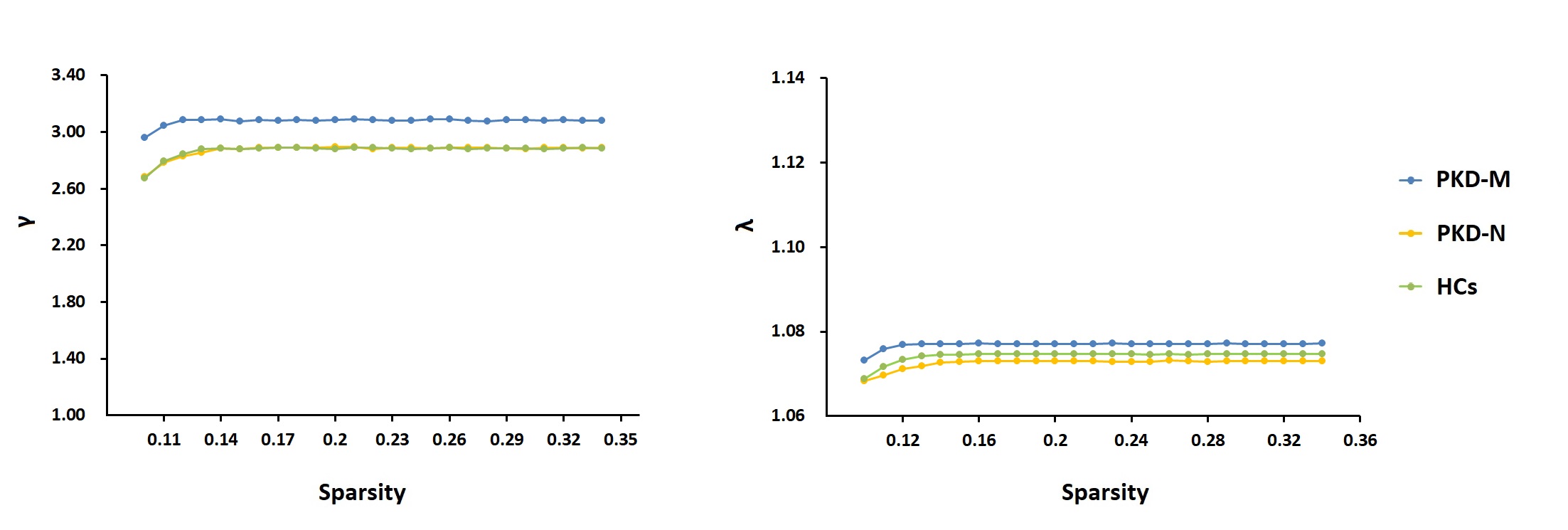


In the defined threshold range, PKD-M, PKD-N and healthy controls exhibited γ substantially larger than 1 and λ approximately equal to 1, indicating that all three groups exhibited the typical features of small-world topology. Abbreviations: HC healthy controls, PKD-M/PKD-N paroxysmal kinesigenic dyskinesia patients with/without PRRT2 mutation.

Figure S3. Comparison of age-related trend differences of nodal efficiency in the left thalamus and left inferior frontal gyrus among PKD-M patients, PKD-N patients, and healthy controls.


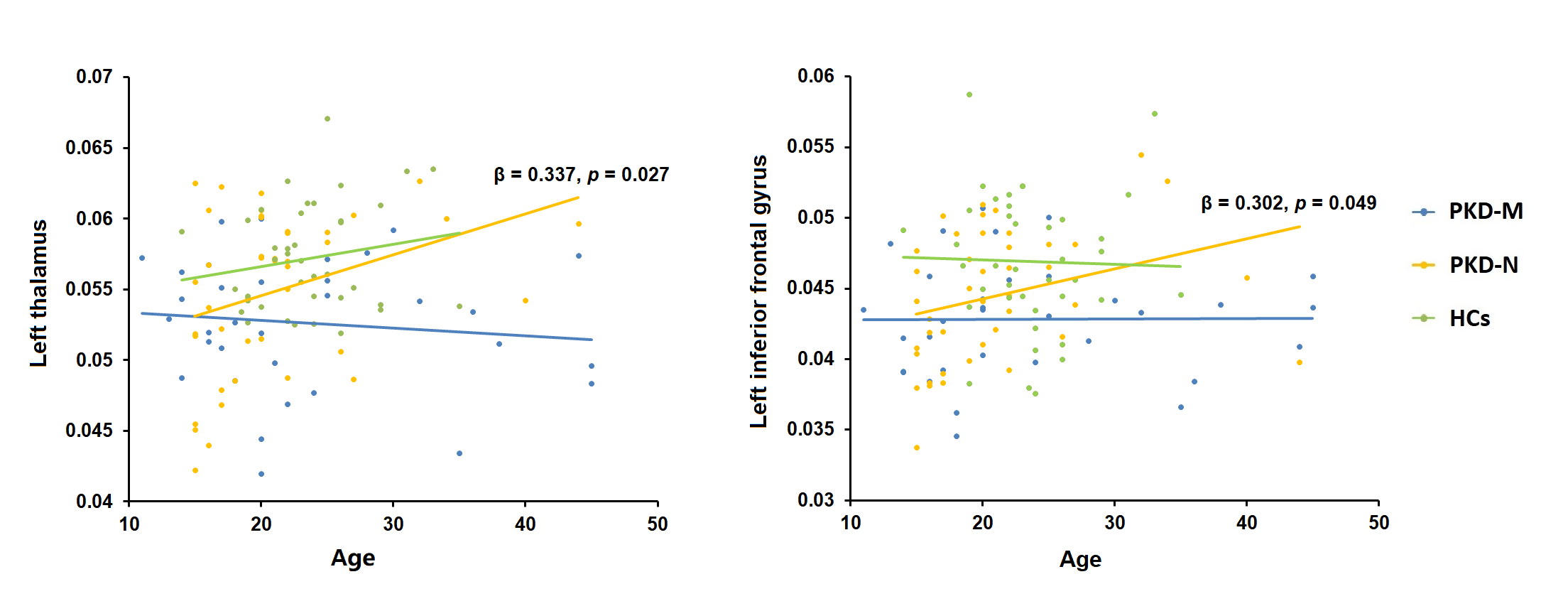


For PKD-N group, the nodal efficiency in the left thalamus and left inferior frontal gyrus increased with age. The age-related differences of nodal efficiency in the left thalamus among three groups were significant (p<0.05). Abbreviations: PKD-M/PKD-N paroxysmal kinesigenic dyskinesia patients with/without PRRT2 mutations, HCs, healthy controls.
